# Impact of motion correction on reproducibility and spatial variability of quantitative myocardial T_2_ mapping

**DOI:** 10.1186/s12968-015-0141-1

**Published:** 2015-06-12

**Authors:** Sébastien Roujol, Tamer A. Basha, Sebastian Weingärtner, Mehmet Akçakaya, Sophie Berg, Warren J. Manning, Reza Nezafat

**Affiliations:** Department of Medicine (Cardiovascular Division), Beth Israel Deaconess Medical Center, 330 Brookline Ave, Boston, MA 02215 USA; Biomedical Engineering Department, Cairo University, Giza, Egypt; Computer Assisted Clinical Medicine, University Medical Center Mannheim, Heidelberg University, Mannheim, Germany; Radiology, Beth Israel Deaconess Medical Center and Harvard Medical School, Boston, MA USA

**Keywords:** Motion correction, Image registration, Quantitative myocardial tissue characterization, Myocardial T_2_ mapping

## Abstract

**Background:**

To evaluate and quantify the impact of a novel image-based motion correction technique in myocardial T_2_ mapping in terms of measurement reproducibility and spatial variability.

**Methods:**

Twelve healthy adult subjects were imaged using breath-hold (BH), free breathing (FB), and free breathing with respiratory navigator gating (FB + NAV) myocardial T_2_ mapping sequences. Fifty patients referred for clinical CMR were imaged using the FB + NAV sequence. All sequences used a T_2_ prepared (T_2_prep) steady-state free precession acquisition. In-plane myocardial motion was corrected using an adaptive registration of varying contrast-weighted images for improved tissue characterization (ARCTIC). DICE similarity coefficient (DSC) and myocardial boundary errors (MBE) were measured to quantify the motion estimation accuracy in healthy subjects. T_2_ mapping reproducibility and spatial variability were evaluated in healthy subjects using 5 repetitions of the FB + NAV sequence with either 4 or 20 T_2_prep echo times (TE). Subjective T_2_ map quality was assessed in patients by an experienced reader using a 4-point scale (1-non diagnostic, 4-excellent).

**Results:**

ARCTIC led to increased DSC in BH data (0.85 ± 0.08 vs. 0.90 ± 0.02, *p* = 0.007), FB data (0.78 ± 0.13 vs. 0.90 ± 0.21, *p* < 0.001), and FB + NAV data (0.86 ± 0.05 vs. 0.90 ± 0.02, *p* = 0.002), and reduced MBE in BH data (0.90 ± 0.40 vs. 0.64 ± 0.19 mm, *p* = 0.005), FB data (1.21 ± 0.65 vs. 0.63 ± 0.10 mm, *p* < 0.001), and FB + NAV data (0.81 ± 0.21 vs. 0.63 ± 0.08 mm, *p* < 0.001). Improved reproducibility (4TE: 5.3 ± 2.5 ms vs. 4.0 ± 1.5 ms, *p* = 0.016; 20TE: 3.9 ± 2.3 ms vs. 2.2 ± 0.5 ms, *p* = 0.002), reduced spatial variability (4TE: 12.8 ± 3.5 ms vs. 10.3 ± 2.5 ms, p < 0.001; 20TE: 9.7 ± 3.5 ms vs. 7.5 ± 1.4 ms) and improved subjective score of T_2_ map quality (3.43 ± 0.79 vs. 3.69 ± 0.55, *p* < 0.001) were obtained using ARCTIC.

**Conclusions:**

The ARCTIC technique substantially reduces spatial mis-alignment among T_2_-weighted images and improves the reproducibility and spatial variability of in-vivo T_2_ mapping.

## Background

The T_2_ relaxation time is dependent on the amount of free water [1] and can be exploited as a potential marker of inflammation and edema [2–7]. In cardiac MR (CMR), T_2_ changes are generally assessed using a dark blood T_2_-weighted acquisition [8]. Elevated signal intensity in T_2_-weighted images have been reported in presence of several cardiomyopathies such as myocarditis [2, 3], Tako-Tsubo [4], and acute myocardial infarction [5–7]. However, this technique only provides qualitative measurements and image interpretation can be limited by several factors including regional signal variations induced by phased array coil, elevated signal induced by sub-endocardial stagnant blood, and signal loss caused by through-plane motion [9, 10].

Quantitative myocardial T_2_ mapping [11, 12] is an alternative technique, which shows promise for reducing uncertainties in interpretations of dark blood T_2_-weighted images. In this technique, several T_2_-weighted images are acquired, each with a different T_2_ contrast. The signal intensity obtained from the T_2_-weighted images is then fit to a physical model of T_2_ signal decay on a per-pixel basis, leading to the creation of a T_2_ map. The acquisition of each T_2_-weighted image was initially performed using either spin echo/fast spin echo acquisitions [11–14] with varying echo times (TE) which results in very long scan time. Recently, T_2_-prepared (T_2_prep) [15] steady-state free precession (SSFP) acquisitions have been proposed and provide higher imaging efficiency [16]. These sequences can be acquired within a breath-hold [16, 17] or under free breathing conditions with respiratory motion correction techniques [16, 18, 19].

Despite the promise of this technique, its in-vivo reproducibility and precision have not been fully characterized. These two factors play a major role for clinical acceptance of any quantitative myocardial tissue characterization technique [20, 21]. The presence of motion among T_2_-weighted images is one of the main challenges in T_2_ mapping and is expected to have important impact on the technique precision and reproducibility.

Breath-hold acquisitions can be used to reduce the impact of respiratory motion. However, some motion can still be detected in 40-60 % of patients due to their limited breath-holding capabilities, as reported by several T_1_ mapping studies using breath-held acquisitions of ~11-17 heart beats [22–25]. The breath-hold approach imposes severe time limitations on the number of acquired T_2_-weighted images (typically ~3-4) since a rest time of ~4-6 heart beats is required between each acquisition to allow for full longitudinal magnetization recovery. Therefore, the use of a free breathing acquisition is attractive as it enables the acquisition of a larger number of T_2_-weighted images which may be beneficial to improve precision and reproducibility. On the other hand, free breathing acquisitions require the use of respiratory navigators to account for through plane motion and image registration algorithms to correct for residual in-plane motion [18].

We recently developed a technique for Adaptive Registration of varying Contrast-weighted images for improved TIssue Characterization (ARCTIC) which we have evaluated for myocardial T_1_ mapping [23]. In this study, we sought to investigate the performance of ARCTIC for T_2_ mapping and its impact on in-vivo reproducibility and spatial variability of myocardial T_2_ estimates.

## Methods

All subjects were scanned using a 1.5 T Philips Achieva (Philips Healthcare, Best, The Netherlands) scanner with a 32-channel cardiac phased array receiver coil. This study was health insurance portability and accountability act (HIPAA) compliant and the imaging protocol was approved by our institutional review board (Committee on Clinical Investigations (CCI)) at the Beth Israel Deaconess Medical Center. Written informed consent was obtained from each participant.

### T_2_ mapping acquisition scheme

T_2_ mapping was performed using our recently reported T_2_ mapping sequence [26] in which multiple T_2_-weighted images are acquired using an electrocardiogram (ECG)-triggered T_2_prep steady-state free precession (SSFP) acquisition with different T_2_prep echo times (TE_T2P_). A rest cycle of 6 s was used between the acquisitions of two successive T_2_-weighted images to ensure full re-growth of the longitudinal magnetization. The TE_T2P_ = 0 image was acquired using 90° pulse followed immediately by a -90° pulse and a crusher gradient to ensure consistency with all other images in term of longitudinal signal reduction induced by imperfect 90° and -90° flip angles used in the T_2_prep. Finally, to model the signal re-growth induced by the SSFP imaging pulses, an infinitely long T_2_prep echo time (TE_T2P_ = ∞) was simulated by acquiring an image immediately after a saturation pulse. In this study, this sequence has been evaluated with 4 T_2_prep echo times (T_2P_4TE: 0, 25, 50, ∞) and 20 T_2_prep echo times (T_2P_20TE: 0, 25, 30, 35, …, 95, 100, ∞, ∞, ∞). For free breathing acquisitions, a respiratory navigator positioned immediately prior to the T_2_prep was used for end expiratory gating (window size = 5 mm). No T_2_prep or imaging pulses were applied if the navigator signal was outside the gating window to enable the acquisition of undisturbed signal in the next heartbeat.

### In-plane motion correction

The ARCTIC approach was used to compensate for in-plane motion between T_2_-weighted images [23]. In this approach, all images are registered individually to a common reference image, which was chosen as the first image of the series (TE_T2P_ = 0). The motion was then estimated in a two-step process. Affine motion descriptors are first estimated over a region of interest surrounding the heart. This global transformation is then provided as input of a more sophisticated local non-rigid motion estimation step using an extended formulation of the optical flow problem which enables the simultaneous estimation of both motion field and intensity variations on a per-pixel basis [27]. An additional term is used to constrain the motion estimates based on prior automatic tracking of specific feature points in the images [28–30]. In this algorithm, both motion field and intensity variation map are solved using an iterative scheme. A multi-resolution approach was used for the local non-rigid motion estimation step where the optical flow is initially estimated from first sub-resolution images and then refined using the full resolution images [31]. For each resolution level, the iterative scheme used 100 iterations and was repeated fifty times. These parameters were empirically optimized in this study. Since optical flow algorithms are well suitable for parallelization on graphic processing unit (GPU) [32–34], a GPU implementation of the method was used based on the compute unified device architecture (CUDA). More details about the algorithm can be found in [23].

### T_2_ map reconstruction

T_2_ maps were reconstructed offline using a 3-parameter curve fitting model.

1 $$ S\left(A,B,{T}_2,{t}_n\right)=A{e}^{-{\mathrm{t}}_n/{T}_2}+B. $$

where t_n_ is the T_2_prep echo time of n^th^ T_2_-weighted image, and A, B, and T_2_ are the model parameters. A, B, and T_2_ are estimated independently for each pixel using a Levenberg-Marquard optimizer with the online library provided in [35].

### In-vivo study in healthy subjects

Twelve healthy adult subjects (32 ± 16 years, 6 male) without any history of cardiovascular disease underwent CMR examination. Each subject was imaged using eight T_2_ mapping sequences in the following order:

1. Breath-held T_2P_4TE
2. Free breathing T_2P_4TE *without* respiratory navigator
3. Free breathing T_2P_4TE *with* respiratory navigator
4. Free breathing T_2P_20TE *with* respiratory navigator (5 repetitions).

All sequences were acquired in the short axis view using a single-shot ECG-triggered acquisition with SSFP imaging readout and the following parameters: field of view = 240 × 240 mm^2^, in-plane resolution = 2.5 × 2.5 mm^2^, slice thickness = 8 mm, TR/TE = 2.7 ms/1.35 ms, flip angle = 85°, 10 linear ramp-up pulses, SENSE rate = 2, acquisition window = 138 ms, number of phase encoding lines = 51, linear k-space ordering. All T_2_ scans were acquired in the same short axis orientation at the mid-diastolic cardiac phase using one single mid-ventricular slice.

Accuracy of motion correction was evaluated in the first three scans (T2p4TE) by quantifying the motion between the T_2_-weighted images without (uncorrected) and with in-plane motion correction using ARCTIC (motion corrected). Endocardial and epicardial contours were manually drawn in all T_2_-weighted images of all T_2_ mapping scans. The two contours were used to create a binary representation of the myocardium for each T_2_-weighted image. The DICE similarity coefficient (DSC) [36] was then calculated between the myocardial binary mask of the reference image (M_ref_) and the myocardial binary mask of each k^th^ T_2_-weighted image (M_k_) as follows:

2 $$ \mathrm{D}\mathrm{S}\mathrm{C}=\frac{2\times \mathrm{area}\left({\mathrm{M}}_{\mathrm{ref}}\cap {\mathrm{M}}_{\mathrm{k}}\right)}{\mathrm{area}\left({\mathrm{M}}_{\mathrm{ref}}\right)+\mathrm{area}\left({\mathrm{M}}_{\mathrm{k}}\right)} $$

The myocardial boundary error (MBE), which provides a local alignment measure is also reported. MBE was measured as the average distance between the myocardial boundary of each T_2_-weighted image (boundary of M_k_) and the myocardial boundary of the reference image (boundary of *M*_*ref*_) as follows:

3 $$ MBE\left({M}_k,{M}_{ref}\right)=\frac{1}{N}{\displaystyle \sum_{i=1}^N\left\Vert {P}_{M_K}^i,-,{P}_{M_{ref}}^{Closest-i}\right\Vert }2 $$

Where ${P}_{M_K}^i$is the i^th^ point along the boundary of M_*k*_, ${P}_{M_{ref}}^{Closest-i}$ is the closest point of ${P}_{M_K}^i$ located on the boundary of *M*_*ref*_. Since TE_T2P_ = ∞ images are very low signal-to-noise ratio (SNR) images, which makes the detection of the myocardial borders very difficult, no DSC/MBE were measured in those images. The statistical significant difference between DSCs (and MBEs) obtained with and without motion correction was evaluated using Wilcoxon signed rank tests. Statistical significance was considered at *p* < 0.05.

The impact of in-plane motion correction on the reproducibility and spatial variability of T_2_ mapping was evaluated using the five T_2P_20TE scans. For each scan, T_2_ maps were reconstructed without (uncorrected) and with prior in-plane motion correction using ARCTIC (motion corrected). The endocardial and epicardial border of the myocardium and the insertion point were manually drawn on each T_2_ map. A six myocardial segment model [37] was automatically created for each single slice (1:anterior, 2:anterospetal, 3:inferospetal, 4:inferior, 5:inferolateral, 6:anterolateral). Segment-based analysis of reproducibility and spatial variability of T_2_ estimates was then performed. Spatial variability was defined as the standard deviation of T_2_ estimates over a given segment. Reproducibility was defined as the standard deviation over the 5 scans of the spatial average T_2_ values in one given segment. Both reproducibility and spatial variability are reported in average over all segments for each subject, and in average over all subjects for each segment. To investigate the motion influence in T_2_ mapping sequences using a limited number of T_2_prep echo times, this overall analysis was repeated using a subset of the T_2_-weighted images from each scan (4 T_2_prep echo times of 0, 25, 50, ∞). The statistical significant difference between uncorrected and ARCTIC motion corrected T_2_ reproducibility (and spatial variability) measured for each subject (in average over all myocardial segments) was evaluated using Wilcoxon signed rank tests.

### In-vivo study in patients

Fifty patients referred for clinical CMR (56 ± 14 y, 29 male) were imaged using the free breathing T_2P_4TE T_2_ mapping sequence with respiratory navigator. All sequences were acquired in the short axis view using a single-shot ECG-triggered acquisition with SSFP imaging readout and the following parameters: field of view = 360 × 360 mm^2^, in-plane resolution = 2 × 2 mm^2^, slice thickness = 8 mm, slice number = 3, TR/TE = 2.9 ms/1.45 ms, flip angle = 85°, 10 linear ramp-up pulses, SENSE rate = 2, acquisition window = 270 ms, number of phase encoding lines = 93, linear k-space ordering. T_2_ maps were reconstructed without and with ARCTIC motion correction.

A subjective qualitative analysis was performed by an experienced cardiologist. The initial motion level in uncorrected data was assessed for each slice as “no motion”, “small motion”, or “large motion” by visual inspection of all uncorrected T_2_-weighted images. Subjective assessment of uncorrected and motion correction T_2_ maps (150 T_2_ maps) followed. Each pair of uncorrected and motion correction T_2_ maps were shown simultaneously to the reader side by side in a random order. The reader was blinded to the reconstruction approach (uncorrected vs. motion corrected). Each map was assessed in term of overall quality (1-non diagnostic/large artifacts/no confidence in interpreting T_2_ values in more than half of the myocardial segments, 2-fair/moderate artifacts/confidence in interpreting T_2_ values in more than half of the myocardial segments, 3-good/small motion artifacts/no confidence in interpreting T_2_ values in at most one myocardial segment, 4-excellent/no motion artifact/confidence in interpreting T_2_ values in all myocardial segments). Furthermore, for each pair of T_2_ maps, the reader was asked to evaluate if any of the two T_2_ map had “1-inferior”, “2-similar”, or “3-superior” quality. Wilcoxon signed rank test was used to test the null hypothesis that the difference of overall T_2_ map quality scores between uncorrected and motion corrected T_2_ maps was zero. Statistical significance was considered at p < 0.05.

## Results

All scans were successful. The nominal scan time (assuming 100% gating efficiency) corresponded to 13 heart beats for the T_2P_4TE sequence and to 99 heart beats for the T_2P_20TE sequence. The employed ARCTIC motion correction and reconstruction of one T_2_ map with 20 T_2_prep echo times was 20s.

**Fig. 1:**
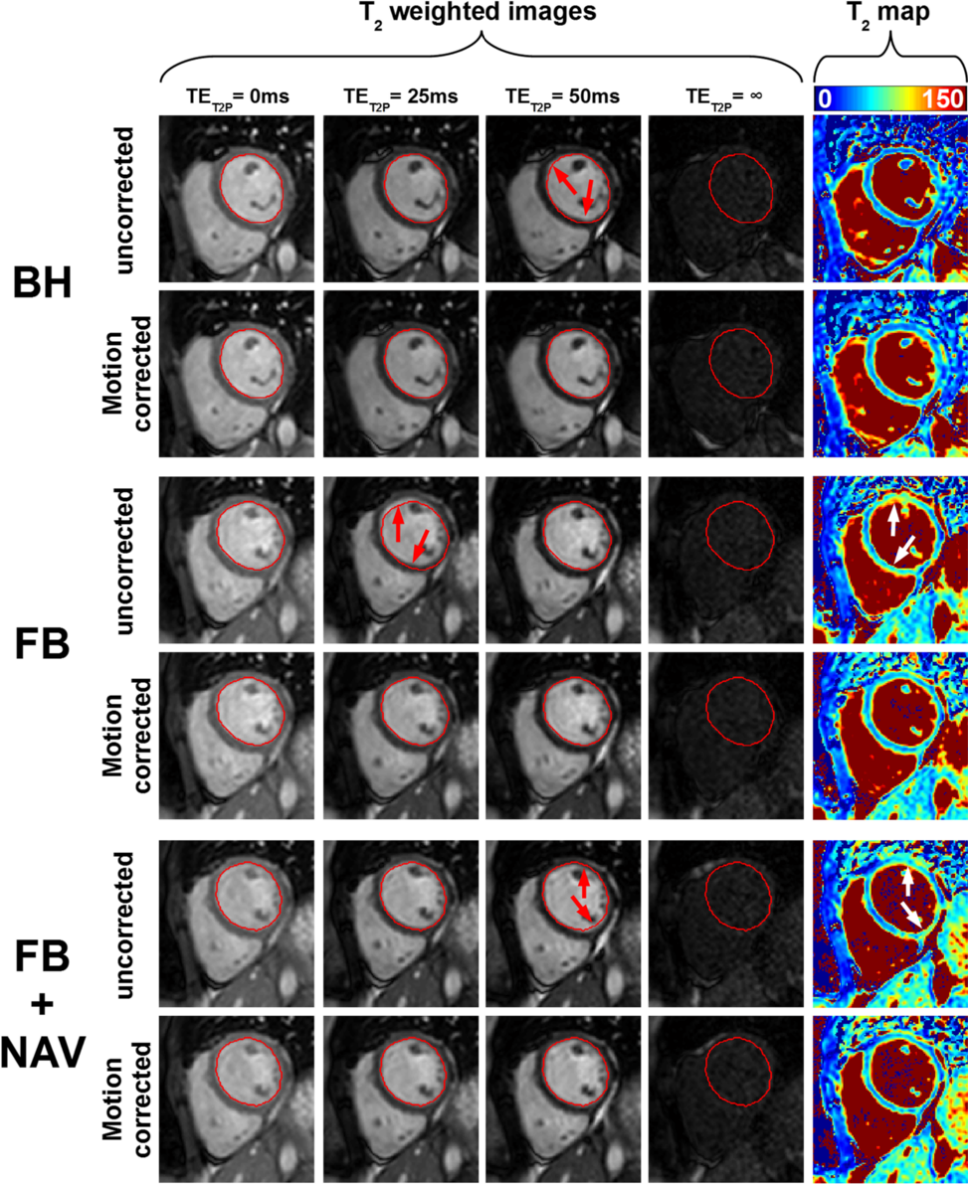
T_2_ scans from one subject acquired using the T_2P_4TE sequence under breath-hold (BH), free breathing (FB), and free breathing with respiratory navigator gating (FB + NAV). Data are shown without (uncorrected) and with (motion corrected) in-plane motion correction. The endocardial contour of the LV myocardium, drawn on the reference image (1^st^ image) of each scan, is reported in all subsequent T_2_-weighted images to facilitate visual motion assessment. Misalignments observed among uncorrected images (red arrows) were substantially reduced after in-plane motion correction using ARCTIC. Furthermore, artifacts in uncorrected T_2_ maps (white arrows) were reduced in motion corrected T_2_ maps

Figure 1 shows an example of the remaining in-plane motion between T_2_-weighted images acquired in one healthy subject using the T_2P_4TE sequence under breath-hold, free breathing, and free breathing with respiratory navigator gating. Motion artifacts can be observed in the reconstructed T_2_ maps (see white arrows). In-plane motion correction improves the spatial alignment of T_2_-weighted images and results in visually improved T_2_ map quality (Figure 1).

**Fig. 2:**
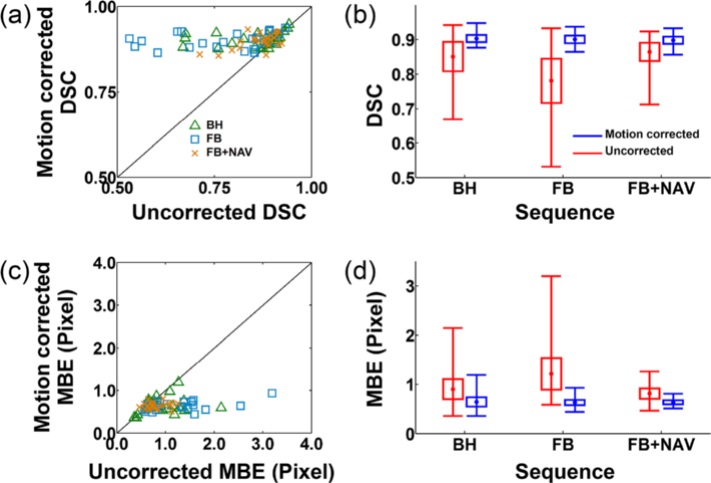
Dice similarity coefficient (DSC) (**a**,**b**) and myocardial boundary error (MBE) (**c**,**d**) obtained using the T_2P_4TE sequence under breath-hold (BH), free breathing (FB), and free breathing with respiratory navigator gating (FB + NAV). DSCs and MBEs of all T_2_-weighted images are shown in (a) and (b), respectively. (**b**) and (**d**) show DSC and MBE as average (central dot), standard deviation (box size) and minimum/maximum (whiskers) over all subjects and all T_2_-weighted images (except the T_2_prep = ∞ images). In-plane motion correction improves the DSC and reduces the MBE for all cases. Furthermore, motion corrected DSC and MBE were similar for all 3 acquisitions (i.e. BH, FB, FB + NAV)

Figure **2** shows quantitative metrics of motion accuracy (DSC and MBE) obtained in healthy subjects using the three aforementioned acquisition sequences. Increased DSC and reduced MBE were observed in each of the three acquisition sequences. In the remaining part of this paragraph, DSC and MBE are reported as (uncorrected data vs. motion corrected data using ARCTIC). On average for all subjects, the DSC increased in breath-hold data (0.85 ± 0.08 vs. 0.90 ± 0.02, *p* = 0.007), free breathing data (0.78 ± 0.13 vs. 0.90 ± 0.21, *p* < 0.001), and free breathing data with respiratory navigator gating (0.86 ± 0.05 vs. 0.90 ± 0.02, *p* = 0.002). The MBE decreased in breath-hold data (0.90 ± 0.40 vs. 0.64 ± 0.19 mm, *p* = 0.005), free breathing data (1.21 ± 0.65 vs. 0.63 ± 0.10 mm, *p* < 0.001), and free breathing data with respiratory navigator gating (0.81 ± 0.21 vs. 0.63 ± 0.08 mm, *p* < 0.001).

**Fig. 3:**
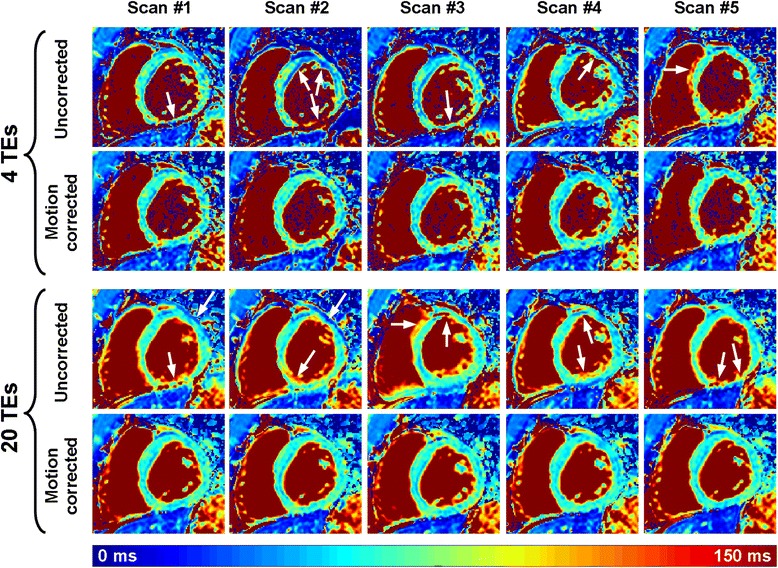
Example of multiple T_2_ maps acquired on the same subject using the T_2P_20TE sequence acquired under free breathing conditions with respiratory navigator gating. T_2_ maps were reconstructed with all T_2_prep echo times (20 TEs) or only a subset of the T_2_prep echo times (0 ms, 25 ms, 50 ms, ∞) (4 TEs). While the remaining in-plane motion generates artifacts on the directly reconstructed T_2_ maps (uncorrected), substantial improvement of T_2_ map quality was obtained using in-plane motion correction (motion corrected). As expected, the homogeneity of the T_2_ maps greatly improved when using all 20 T_2_prep echo times compared to only 4 T_2_prep echo times

Figure **3** shows an example of multiple T_2_ maps obtained in one healthy subject using the T_2P_20TE sequence acquired under free breathing conditions with respiratory navigator gating. T_2_ maps are shown when reconstructed from only 4 T_2_prep echo times and from all 20 T_2_prep echo times. The level of artifacts in uncorrected T_2_ maps appears higher than in motion corrected T_2_ maps (see white arrows). As expected, motion artifact patterns have high spatial variability in uncorrected T_2_ maps. Furthermore, the spatial variability of the myocardial T_2_ estimates appears well reduced when using all 20 T_2_prep echo times compared to only 4 T_2_prep echo times.

**Fig. 4:**
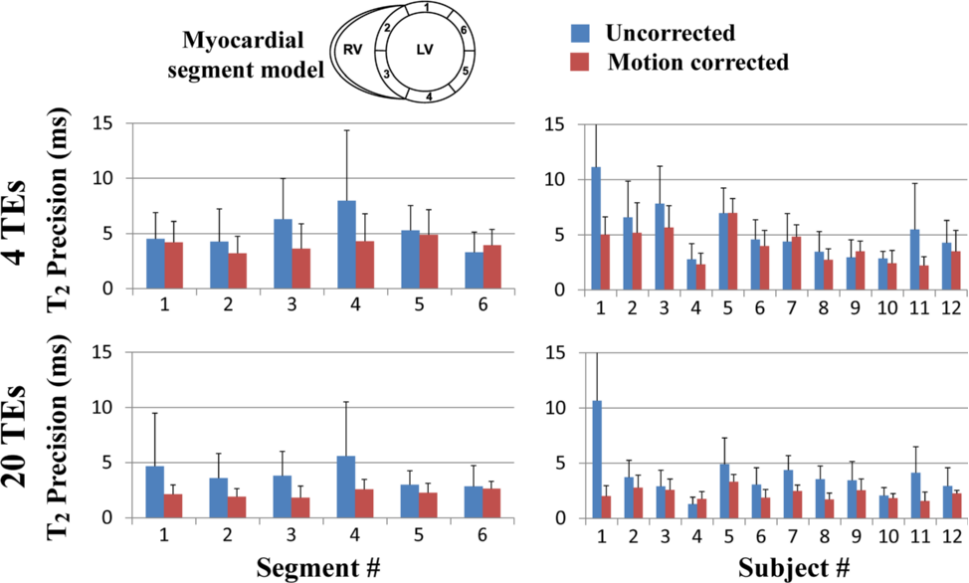
Reproducibility of T_2_ mapping using the T_2P_20TE sequence acquired under free breathing with respiratory navigator gating. Reproducibility was evaluated for T_2_ maps reconstructed using only a subset of the T_2_prep echo times (0 ms, 25 ms, 50 ms, ∞) (4TEs) (**a**,**b**) and using all 20 T_2_prep echo times (20 TEs) (**c**,**d**). Average and standard deviation of T_2_ reproducibility is reported over all subjects for each segment (**a**,**c**) and over all segments for each subject (**b**,**d**). In-plane motion correction using ARCTIC improves the reproducibility of T_2_ mapping

**Fig. 5:**
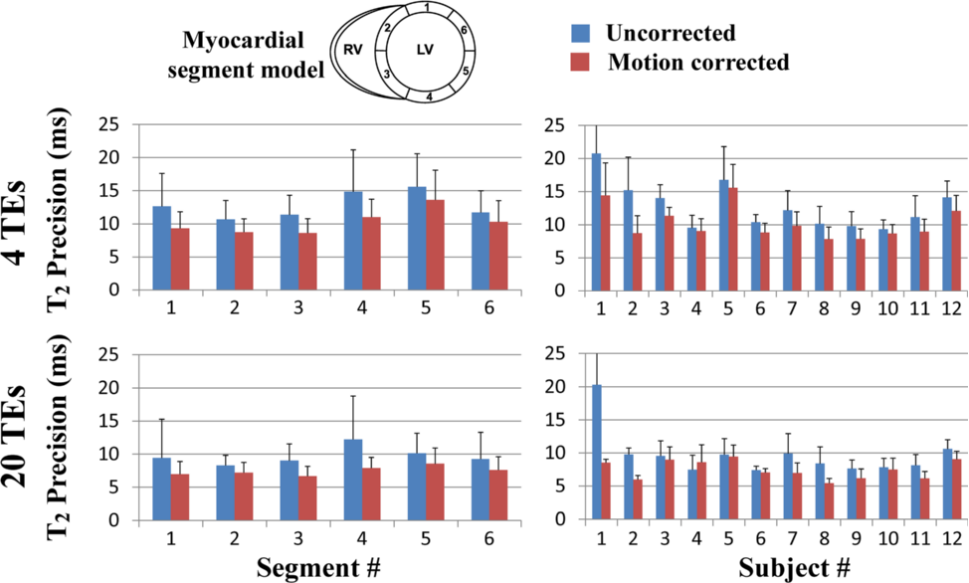
Spatial variability of T_2_ mapping in all subjects using the T_2P_20TE sequence acquired under free breathing conditions with respiratory navigator gating. Spatial variability was evaluated for T_2_ maps reconstructed using only a subset of the T_2_prep echo times (0 ms, 25 ms, 50 ms, ∞) (4TEs) (**a**,**b**) and using all 20 T_2_prep echo times (20 TEs) (**c**,**d**). Average and standard deviation of T_2_ spatial variability is reported over all subjects for each segment (**a**,**c**) and over all segments for each subject (**b**,**d**). In-plane motion correction using ARCTIC reduced the spatial variability of T_2_ mapping

Figures 4 and 5 summarize the reproducibility and spatial variability of T_2_ measurements obtained in healthy subjects using the T_2P_20TE sequence. Results are shown for uncorrected and motion corrected T_2_ maps reconstructed using either 4 T_2_prep echo times or 20 T_2_prep echo times. Reproducibility and spatial variability are reported as uncorrected T_2_ maps vs. motion corrected T_2_ maps using ARCTIC. Improved reproducibility was observed over all subjects and myocardial segments in T_2_ maps reconstructed from 4 T_2_prep echo times (5.3 ± 2.5 ms vs. 4.0 ± 1.5 ms, *p* = 0.016) and 20 T_2_prep echo times (3.9 ± 2.3 ms vs. 2.2 ± 0.5 ms, *p* = 0.002). Similarly, reduced spatial variability was observed over all subjects and myocardial segments in T_2_ maps reconstructed from 4 T_2_prep echo times (12.8 ± 3.5 ms vs. 10.3 ± 2.5 ms, *p* < 0.001) and 20 T_2_prep echo times (9.7 ± 3.5 ms vs. 7.5 ± 1.4 ms, *p* = 0.005).

As expected, T_2_ maps reconstructed using 20 T_2_prep echo times had better reproducibility than those reconstructed using only 4 T_2_prep echo times in both uncorrected data (3.9 ± 2.3 ms vs. 5.3 ± 2.5 ms, respectively, *p* = 0.007) and motion corrected (2.2 ± 0.5 ms vs. 4.0 ± 1.5 ms, respectively, *p* < 0.001). The spatial variability of myocardial T_2_ estimates reconstructed using 20 T_2_prep echo times was also lower than the one obtained with 4 T_2_prep echo times in both uncorrected data (9.7 ± 3.5 ms vs. 12.8 ± 3.5 ms, respectively, *p* < 0.001) and motion corrected data (7.5 ± 1.4 ms vs. 10.3 ± 2.5 ms, respectively, *p* < 0.001).

**Fig. 6:**
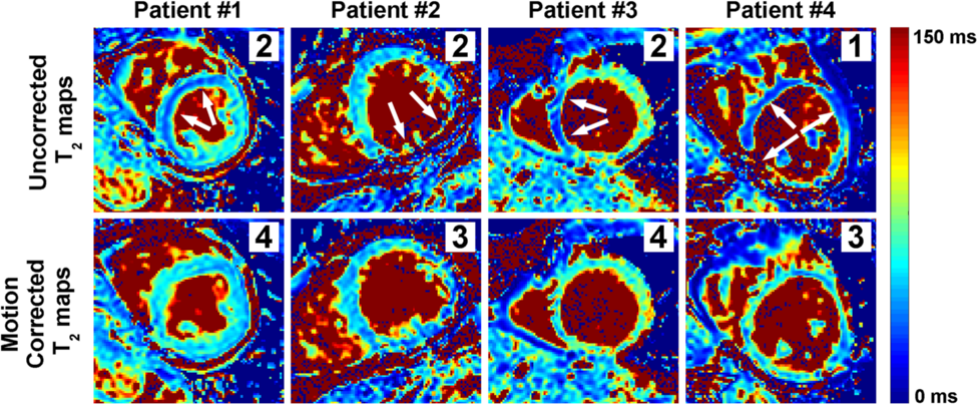
Example uncorrected and ARCTIC motion corrected T_2_ maps obtained in patients. Subjective T_2_ map quality scores are shown for each map (right upper corner). Motion among T_2_-weighted resulted in large regional variations/artifacts in myocardial T_2_ estimates of uncorrected maps (see white arrows) and were substantially reduced using the proposed ARCTIC motion correction

Figure 6 shows example uncorrected and ARCTIC motion corrected T_2_ maps obtained in four patients. Large regional variations and artifacts can be observed in uncorrected T_2_ maps (see white arrows). The proposed ARCTIC motion correction substantially improved the T_2_ map quality in all 4 patients.

**Fig. 7:**
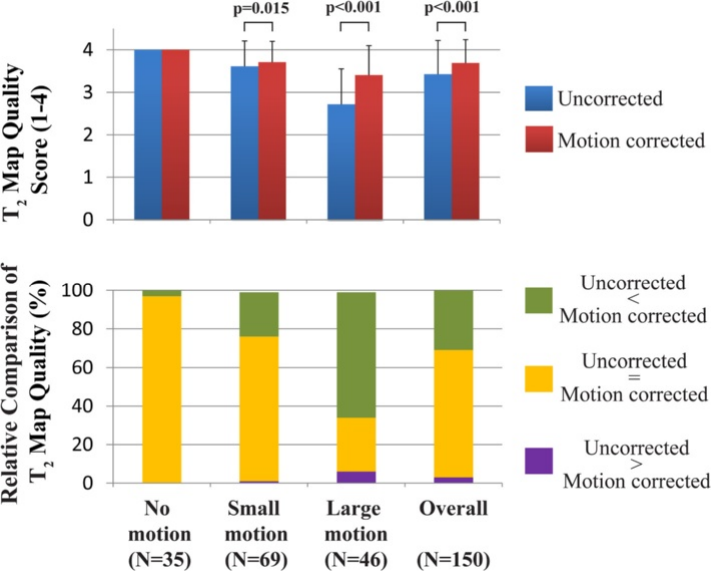
Subjective assessment of T_2_ map quality in patients. In-plane motion correction using ARCTIC increased T_2_ map quality scores (3.69 ± 0.55 vs. 3.43 ± 0.79, *p* < 0.001)

Figure **7** shows the subjective assessment of T_2_ map quality obtained in 50 patients. Overall (*N* = 150 T_2_ maps), ARCTIC motion corrected T_2_ maps had higher quality score than uncorrected T_2_ maps (3.69 ± 0.55 vs. 3.43 ± 0.79, *p* < 0.001). In the relative comparison of T_2_ map quality, uncorrected T_2_ maps has superior, similar, and inferior quality than ARCTIC motion corrected T_2_ maps in 4 maps (3 %), 99 maps (66 %), and 47 maps (31 %), respectively. Furthermore, the motion level was assessed as “no motion” in 35 slices (23%), “small motion” in 69 slices (46%), and “large motion” in 46 slices (30%). In “no motion” data, all ARCTIC motion corrected and uncorrected T_2_ maps received a subjective quality score of 4.0 and 97 % of them had similar relative quality. In “small motion” data, ARCTIC motion corrected T_2_ maps had higher subjective quality score (3.71 ± 0.49 vs. 3.61 ± 0.60, *p* = 0.015) and superior (23%), similar (75%) and inferior (1%) relative quality than uncorrected T_2_ maps. In “large motion” data, ARCTIC motion corrected T_2_ maps had higher subjective quality score (3.41 ± 0.69 vs. 2.72 ± 0.83, *p* < 0.001) and superior (65%), similar (28%) and inferior (6%) relative quality than uncorrected T_2_ maps.

## Discussion

In this study, we demonstrate the benefit of in-vivo in-plane ARCTIC motion correction in myocardial T_2_ mapping. The method provides improved alignment of the myocardium in T_2_-weighted images acquired with breath-hold acquisitions and free breathing acquisitions with and without respiratory navigator gating. ARCTIC motion correction improves T_2_ map quality which results in improved reproducibility and spatial variability of myocardial T_2_ estimates. Finally, the CPU/GPU implementation of ARCTIC substantially reduces the computation time of the T_2_ map reconstruction to 20s which is suitable for clinical applicability.

DSCs and MBEs found in this study are in good agreement with previous studies [23–25, 38]. As expected higher mis-alignments were observed using free breathing acquisitions without respiratory navigator gating. DSCs/MBEs improvement was obtained in all three types of acquisitions. This confirms the benefit of motion correction, even for data acquired with a breath-hold. This is likely because 40-60% of patients fail to sustain a stable breath-hold in these conditions [23–25]. Furthermore, similar DSCs/MBEs were obtained after motion correction using the three acquisition conditions (breath-hold and free breathing with and without respiratory navigator). It is important to note that through-plane motion cannot be compensated when using the free breathing acquisition without respiratory navigator gating. In this case, the efficacy of in-plane motion correction algorithms depends on the subject’s heart orientation in relation to his respiratory movement. The use of respiratory navigator appears thus desirable to enable through plane motion compensation in free breathing acquisitions. The registration accuracy was not evaluated in the TE_T2P_ = ∞ images since the contrast is too low to identify the myocardium. Motion correction is expected to have slightly lower accuracy in those images due to the expected limited ability to compensate for complex motion.

The ARCTIC approach successfully corrected the encountered motion in all subjects. In this study, the heart motion patterns were mainly influenced by the breathing activity of the subjects and to lesser extent to their RR-interval variations. However, the motion pattern can be more complex in patients imaged during arrhythmic events. The performance of the method in such conditions was not investigated and should be addressed in future work.

The reproducibility and spatial variability of T_2_ mapping was improved using ARCTIC. The use of 20 T_2_-weighted images improved the reproducibility and the spatial variability of T_2_ mapping (over the use of only 4 T_2_-weighted images) by a factor of 2 and 1.4, respectively. Therefore, the choice of the number of T_2_prep echo times depends on the desired trade-off between acquisition time and T_2_ map quality. Further studies are warranted to determine the clinically relevant threshold providing satisfactory T_2_ map quality in a reasonable amount of time.

Reproducibility and spatial variability of T_2_ estimates were found similar in all myocardial segments when using 20 TEs. However, slight differences seemed to be observed when using 4TEs only, especially in the myocardial segment #4 (inferior wall). Several factors could have contributed to this observation including 1) increased sensitivity to cardiac motion and partial voluming in the free wall due to reduced wall thickness, 2) increased field inhomogeneity in myocardial segments located at the heart/lung interface. Future studies are warranted to study the impact of each of these factors.

In this study, the data were acquired using our recently developed T_2_ mapping sequence. The ARCTIC approach is expected to provide similar motion correction performance using other T_2_ mapping sequences. Nevertheless, the impact of motion correction on the reproducibility and spatial variability of other T_2_ mapping sequences may be different and is beyond the scope of this study. Furthermore, all data were acquired in 2D. 3D myocardial T_2_ mapping may represent a valuable approach for true 3D assessment of pathological tissues [39, 40]. The extension of the ARCTIC approach to 3D is straightforward and is expected to provide similar improvement of the reproducibility and spatial variability of 3D T_2_ mapping.

There are several limitations in this study. In the in-vivo analysis of reproducibility and spatial variability, the 4TEs data were extracted from the T_2P_20TE sequence and were thus not acquired using the T_2P_4TE sequence. However, since the T_2P_20TE sequence was acquiring with respiratory gating, the potential bias in reproducibility and spatial variability obtained in the 4TEs data should have been kept to the minimum. Finally, the study was only performed in healthy adult subjects with limited sample size. Further studies are warranted to confirm the benefit of the ARCTIC approach to improve the reproducibility and spatial variability of myocardial T_2_ mapping in patients.

## Conclusions

The ARCTIC technique substantially reduces spatial mis-alignment among T_2_-weighted images. This method improves the reproducibility and reduces the spatial variability of in-vivo T_2_ mapping. Furthermore, the in-vivo reproducibility and spatial variability of T_2_ mapping is improved using a higher number of T_2_prep echo times combined with ARCTIC motion correction.

## Abbreviations

T2prep: T2 prepared
ARCTIC: Adaptive registration of varying contrast-weighted images for improved tissue characterization
BH: Breath-hold
FB: Free breathing
FBNAV: Free breathing conditions with respiratory navigator gating
DSC: DICE similarity coefficient
MBE: Myocardial boundary errors (MBE)
TE: Eecho times
SSFP: Steady-state free precession
HIPAA: Health insurance portability and accountability act
ECG: Electrocardiogram
TET2P: T2prep echo times
TR: Repetition time
FOV: Field of view
SENSE: Sensitivity encoding
SNR: Signal-to-noise ratio
GPU: Graphic processing unit
CUDA: Compute unified device architecture

## References

[1] Higgins CB, Herfkens R, Lipton MJ, Sievers R, Sheldon P, Kaufman L (1983). Nuclear magnetic resonance imaging of acute myocardial infarction in dogs: alterations in magnetic relaxation times. Am J Cardiol.

[2] Abdel-Aty H, Boye P, Zagrosek A, Wassmuth R, Kumar A, Messroghli D (2005). Diagnostic performance of cardiovascular magnetic resonance in patients with suspected acute myocarditis: comparison of different approaches. J Am Coll Cardiol.

[3] Friedrich MG, Sechtem U, Schulz-Menger J, Holmvang G, Alakija P, Cooper LT (2009). Cardiovascular magnetic resonance in myocarditis: A JACC White Paper. J Am Coll Cardiol.

[4] Abdel-Aty H, Cocker M, Friedrich MG (2009). Myocardial edema is a feature of Tako-Tsubo cardiomyopathy and is related to the severity of systolic dysfunction: insights from T2-weighted cardiovascular magnetic resonance. Int J Cardiol.

[5] Abdel-Aty H, Zagrosek A, Schulz-Menger J, Taylor AJ, Messroghli D, Kumar A (2004). Delayed enhancement and T2-weighted cardiovascular magnetic resonance imaging differentiate acute from chronic myocardial infarction. Circulation.

[6] Raman SV, Simonetti OP, Winner MW, Dickerson JA, He X, Mazzaferri EL (2010). Cardiac magnetic resonance with edema imaging identifies myocardium at risk and predicts worse outcome in patients with non-ST-segment elevation acute coronary syndrome. J Am Coll Cardiol.

[7] Cury RC, Shash K, Nagurney JT, Rosito G, Shapiro MD, Nomura CH (2008). Cardiac magnetic resonance with T2-weighted imaging improves detection of patients with acute coronary syndrome in the emergency department. Circulation.

[8] Simonetti OP, Finn JP, White RD, Laub G, Henry DA (1996). "Black blood" T2-weighted inversion-recovery MR imaging of the heart. Radiology.

[9] Abdel-Aty H, Simonetti O, Friedrich MG (2007). T2-weighted cardiovascular magnetic resonance imaging. J Magn Reson Imaging.

[10] Arai AE (2008). Using magnetic resonance imaging to characterize recent myocardial injury: utility in acute coronary syndrome and other clinical scenarios. Circulation.

[11] Bottomley PA, Foster TH, Argersinger RE, Pfeifer LM (1984). A review of normal tissue hydrogen NMR relaxation times and relaxation mechanisms from 1-100 MHz: dependence on tissue type, NMR frequency, temperature, species, excision, and age. Med Phys.

[12] McNamara MT, Higgins CB, Schechtmann N, Botvinick E, Lipton MJ, Chatterjee K (1985). Detection and characterization of acute myocardial infarction in man with use of gated magnetic resonance. Circulation.

[13] Foltz WD, Stainsby JA, Wright GA (1997). T2 accuracy on a whole-body imager. Magn Reson Med.

[14] He T, Gatehouse PD, Anderson LJ, Tanner M, Keegan J, Pennell DJ (2006). Development of a novel optimized breathhold technique for myocardial T2 measurement in thalassemia. J Magn Reson Imaging.

[15] Brittain JH, Hu BS, Wright GA, Meyer CH, Macovski A, Nishimura DG (1995). Coronary angiography with magnetization-prepared T2 contrast. Magn Reson Med.

[16] Huang TY, Liu YJ, Stemmer A, Poncelet BP (2007). T2 measurement of the human myocardium using a T2-prepared transient-state TrueFISP sequence. Magn Reson Med.

[17] Giri S, Chung YC, Merchant A, Mihai G, Rajagopalan S, Raman SV (2009). T2 quantification for improved detection of myocardial edema. J Cardiovasc Magn Reson.

[18] Giri S, Shah S, Xue H, Chung YC, Pennell ML, Guehring J (2012). Myocardial T(2) mapping with respiratory navigator and automatic nonrigid motion correction. Magn Reson Med.

[19] Blume U, Lockie T, Stehning C, Sinclair S, Uribe S, Razavi R (2009). Interleaved T(1) and T(2) relaxation time mapping for cardiac applications. J Magn Reson Imaging.

[20] Kellman P, Hansen MS (2014). T1-mapping in the heart: accuracy and precision. J Cardiovasc Magn Reson.

[21] Piechnik SK, Ferreira VM, Lewandowski AJ, Ntusi NA, Banerjee R, Holloway C (2013). Normal variation of magnetic resonance T1 relaxation times in the human population at 1.5 T using ShMOLLI. J Cardiovasc Magn Reson.

[22] Cheng ASH, Pegg TJ, Karamitsos TD, Searle N, Jerosch-Herold M, Choudhury RP (2007). Cardiovascular magnetic resonance perfusion imaging at 3-tesla for the detection of coronary artery disease. J Am Coll Cardiol.

[23] Roujol S, Foppa M, Weingartner S, Manning WJ, Nezafat R. Adaptive registration of varying contrast-weighted images for improved tissue characterization (ARCTIC): application to T1 mapping. Magn Reson Med. 2015;73(4):1469-82. 10.1002/mrm.25270 PMC4221574 24798588

[24] Xue H, Greiser A, Zuehlsdorff S, Jolly MP, Guehring J, Arai AE (2013). Phase-sensitive inversion recovery for myocardial T1 mapping with motion correction and parametric fitting. Magn Reson Med.

[25] Xue H, Shah S, Greiser A, Guetter C, Littmann A, Jolly MP (2012). Motion correction for myocardial T1 mapping using image registration with synthetic image estimation. Magn Reson Med.

[26] Akçakaya M, Basha TA, Weingärtner S, Roujol S, Berg S, Nezafat R. Improved quantitative myocardial T2 mapping. Mag Reson Med 2014, In Press. doi:10.1002/mrm.25377. 10.1002/mrm.25377 PMC4320682 25103908

[27] Cornelius N, Kanade T (1984). Adapting optical-flow to measure object motion in reflectance and X-ray image sequences. ACM SIGGRAPH Comput Graph.

[28] Butler CR, Thompson R, Haykowsky M, Toma M, Paterson I (2009). Cardiovascular magnetic resonance in the diagnosis of acute heart transplant rejection: a review. J Cardiovasc Magn Reson.

[29] Roujol S, Benois-Pineau J, de Senneville BD, Quesson B, Ries M, Moonen C. Real time constrained motion estimation for ECG-gated cardiac MRI. 2010. IEEE. p 757-760.

[30] Roujol S, Benois-Pineau J, de Senneville BD, Ries M, Quesson B, Moonen CT (2012). Robust real-time-constrained estimation of respiratory motion for interventional MRI on mobile organs. IEEE Trans Inf Technol Biomed.

[31] Pratikakis I, Barillot C, Hellier P, Memin E (2003). Robust multiscale deformable registration of 3D ultrasound images. International Journal of Image and Graphics.

[32] de Senneville BD, Noe KO, Ries M, Pedersen M, Moonen CT, Sorensen T. An optimised multi-baseline approach for on-line MR-temperature monitoring on commodity graphics hardware. 2008. IEEE. p 1513-1516.

[33] Ostergaard Noe K, De Senneville BD, Elstrom UV, Tanderup K, Sorensen TS (2008). Acceleration and validation of optical flow based deformable registration for image-guided radiotherapy. Acta Oncol.

[34] Roujol S, Ries M, Quesson B, Moonen C, Denis de Senneville B (2010). Real-time MR-thermometry and dosimetry for interventional guidance on abdominal organs. Magn Reson Med.

[35] Lourakis M. levmar: Levenberg-Marquardt nonlinear least squares algorithms in C/C++. www.ics.forth.gr/~lourakis/levmar/ 2004, Updated on November 29, 2011, Accessed on June 1, 2013.

[36] Dice LR (1945). Measures of the amount of ecologic association between species. Ecology.

[37] Cerqueira MD, Weissman NJ, Dilsizian V, Jacobs AK, Kaul S, Laskey WK (2002). Standardized myocardial segmentation and nomenclature for tomographic imaging of the heart. A statement for healthcare professionals from the Cardiac Imaging Committee of the Council on Clinical Cardiology of the American Heart Association Circulation.

[38] Cheng C, Herfkens R, Taylor C (2003). Inferior vena caval hemody- namics quantified in vivo at rest and during cycling exercise using magnetic resonance imaging. Am J Physiol Heart Circ Physiol.

[39] Ding H, Schär M, Zviman M, Halperin HR, Beinart R, Herzka DA (2013). High-resolution quantitative 3D T2 mapping allows quantification of changes in edema after myocardial infarction. J Cardiovasc Magn Reson.

[40] Heeswijk RB, Piccini D, Feliciano H, Hullin R, Schwitter J, Stuber M. Self-navigated isotropic three-dimensional cardiac T2 mapping. Magn Reson Med. 2015;73(4):1549-54. 10.1002/mrm.25258 24809849

